# Development of a low-cost cellulase production process using *Trichoderma reesei* for Brazilian biorefineries

**DOI:** 10.1186/s13068-017-0717-0

**Published:** 2017-02-02

**Authors:** Simo Ellilä, Lucas Fonseca, Cristiane Uchima, Junio Cota, Gustavo Henrique Goldman, Markku Saloheimo, Vera Sacon, Matti Siika-aho

**Affiliations:** 1 VTT Brasil Ltda., Barueri, Sao Paulo Brazil; 2 VTT Technical Research Centre of Finland, Tietotie 2, 02044 Espoo, Finland; 3 Instituto de Ciências Agrárias, Universidade Federal de Minas Gerais (UFMG), Montes Claros, MG Brazil; 4 Faculdade de Ciências Farmacêuticas de Ribeirão Preto, Universidade de Sao Paulo, Ribeirão Preto, SP Brazil

**Keywords:** On-site, Cellulase, Enzyme, *Trichoderma reesei*, Sugarcane, Molasses, Soybean hulls, Brazil, Biorefinery, Cellulosic ethanol

## Abstract

**Background:**

During the past few years, the first industrial-scale cellulosic ethanol plants have been inaugurated. Although the performance of the commercial cellulase enzymes used in this process has greatly improved over the past decade, cellulases still represent a very significant operational cost. Depending on the region, transport of cellulases from a central production facility to a biorefinery may significantly add to enzyme cost. The aim of the present study was to develop a simple, cost-efficient cellulase production process that could be employed locally at a Brazilian sugarcane biorefinery.

**Results:**

Our work focused on two main topics: growth medium formulation and strain improvement. We evaluated several Brazilian low-cost industrial residues for their potential in cellulase production. Among the solid residues evaluated, soybean hulls were found to display clearly the most desirable characteristics. We engineered a *Trichoderma reesei* strain to secrete cellulase in the presence of repressing sugars, enabling the use of sugarcane molasses as an additional carbon source. In addition, we added a heterologous β-glucosidase to improve the performance of the produced enzymes in hydrolysis. Finally, the addition of an invertase gene from *Aspegillus niger* into our strain allowed it to consume sucrose from sugarcane molasses directly. Preliminary cost analysis showed that the overall process can provide for very low-cost enzyme with good hydrolysis performance on industrially pre-treated sugarcane straw.

**Conclusions:**

In this study, we showed that with relatively few genetic modifications and the right growth medium it is possible to produce considerable amounts of well-performing cellulase at very low cost in Brazil using *T. reesei*. With further enhancements and optimization, such a system could provide a viable alternative to delivered commercial cellulases.

**Electronic supplementary material:**

The online version of this article (doi:10.1186/s13068-017-0717-0) contains supplementary material, which is available to authorized users.

## Background

Lignocellulosic biomass represents perhaps the only viable renewable alternative to petroleum as a raw material for the production of fuels and chemicals in the future. Lignocellulosic biomass is available in abundance in side streams of the agricultural and forest industries across the globe. Converting lignocellulosic biomass into fuels and chemicals along the standard biochemical route entails a physicochemical pre-treatment of the biomass, followed by enzymatic hydrolysis of the polysaccharide components cellulose and hemicellulose into monomeric sugars. These sugars can then be further fermented into ethanol or other desired compounds.

Although commercial cellulases have improved significantly over the past decade, enzymes remain a significant cost factor in the cellulosic ethanol process [1]. Enzymes can present a particular hurdle in some biomass rich countries such as Brazil, where no domestic industrial cellulase production exists and transport infrastructure can be limiting. The industrial production of cellulase enzymes is performed by fermenting highly developed strains of filamentous ascomycete fungi, expertise mainly held by a handful of American and European companies [2].

Several authors have previously discussed the possibility of circumventing the costs associated with enzyme transport by producing the enzymes in a distributed manner at their final site of use (“on-site” enzyme production) [3–6]. As the enzyme would not be transported, cost-savings could be achieved by avoiding process steps such as enzyme clarification and stabilization, and using whole fungal fermentation broth in hydrolysis instead [7, 8]. It is often envisioned that crude raw materials, perhaps the lignocellulosic biomass itself, could be used as the raw material for enzyme production [1, 4, 6, 9–12] and thus significantly lower the cost of the enzymes. Detailed techno-economic modeling has indeed suggested that the carbon source used in enzyme production could account for more than 50% of the total enzyme cost, if it were pure glucose [5]. Based on the same model, the cost of enzyme ($/kg) was found to dramatically impact the minimum ethanol selling price (MESP) of the cellulosic ethanol process [13].

The most common organism cited for the production of cellulases is the mesophilic filamentous ascomycete fungus *Trichoderma reesei* [14]. Industrial strains and processes have been reported to reach enzyme titers in excess of 100 g/l [15]. However, the induction of high-level cellulase production in conventional *T. reesei* strains is dependent on inducers such as pure cellulose, lactose or sophorose [9, 16], costly media components that would likely render the produced enzymes too expensive for a cellulosic ethanol process.

Furthermore, the secretomes of conventional *T. reesei* strains generally lack sufficient β-glucosidase [17] and hemicellulase [11] activities for the enzymes to perform well in the hydrolysis of pre-treated biomass. In biomass hydrolysis studies, it has therefore been common to combine *T. reesei* culture supernatants with enzymes from other fungi secreting higher levels of these enzymes, typically *Aspergillus* spp. [8, 10, 11, 18, 19]. However, for a simplified low-cost on-site cellulase production process it would be highly desirable to produce all required enzymes from a single host and process. Previous work from several authors suggests ways around the aforementioned problems hampering the use of *T. reesei* as an on-site cellulase producer.

*Trichoderma reesei* could be modified to produce more enzymes and perhaps on lower cost carbon sources. The primary targets for such modifications would be the transcription factors controlling the production of cellulases. Several transcription factors relevant in this context have been described in *T. reesei*, such as CRE1, ACE1, ACE2, HAP2/3/5, XYR1, [20] and more recently others [21]. The expression patterns of some of these transcription factors are already altered in hypercellulolytic strains of *T. reesei* [20]. The expression level of the transcription factor *xyr1* seems to be most directly correlated with the expression levels of the main (hemi)cellulases produced by *T. reesei* [22]. Indeed, the overexpression of this transcription factor has been found to lead to increased cellulase production in *T. reesei* Rut-C30 [23, 24]. Additionally, this transcription factor appears to be involved in the repression of enzyme production on glucose, with a particular mutation (A824V) being able to abolish this repressive function [25]. Similar results were previously seen with a valine to phenylalanine mutation in the same region of the *A. niger* homologue (*xlnR*) of this transcription factor [26]. The residue at this position is conserved in the *T. reesei* transcription factor (V821). Additional gains in enzyme production by Rut-C30 were seen by down-regulating the repressor *ace1* using RNA interference [23].

Several studies have also addressed the main drawback of *T. reesei* secretomes, namely the lack of sufficient β-glucosidase activity. The lack of β-glucosidase leads to the accumulation of cellobiose during hydrolysis, which in turn slows down the activity of the other key cellulases such as cellobiohydrolases and endoglucanases. *T. reesei* strains have been engineered to overexpress native [27, 28] and heterologous [29–33] β-glucosidases in several prior studies.

In the present study, we aimed to develop a simple cellulase production system based on the filamentous fungus *T. reesei* that could be operated at a Brazilian sugarcane biorefinery. We considered various industrial residues available in Brazil and used them to formulate a simple low-cost culture medium. Additionally, we engineered our production strain to secrete enzymes in the presence of repressing sugars and added a heterologous β-glucosidase from *Talaromyces emersonii* to improve the performance of the produced enzymes in hydrolysis. A further addition of an invertase gene from *A. niger* into our strain allowed it to consume sucrose from sugarcane molasses directly, removing the necessity to invert the sucrose using acid or other means.

## Results

### Selection of soybean hulls as a carbon source for cellulase production

Modeling has shown that the primary carbon source used for enzyme production could account for over 50% of the cost of the final enzyme [5]. We therefore initially aimed to identify industrial residues that could be used in the formulation of a low-cost *T. reesei* culture medium. Ideally, such a residue should be available in abundance at low cost, display good rheological properties (i.e., low viscosity), be non-toxic and have high nutrient availability to the enzyme-producing fungus, and induce cellulase production. In the most ideal case, the carbon source would be available at the cellulosic ethanol plant. At a Brazilian sugarcane biorefinery, this could mean *in*-*natura* or pre-treated sugarcane bagasse or straw, sugarcane juice, or molasses. However, as we tested these raw materials with unsatisfactory results, we broadened our scope to industrial residues in general. The evaluated residues are listed in Additional file 1, along with our observations regarding the aforementioned factors of price, availability, rheology, toxicity, and enzyme production potential.

Soybean hulls emerged as an excellent residue due to a unique combination of properties based on this simple evaluation. Not only is this residue relatively cheap ($100–120/t) and available in abundance in Brazil, it contributes to medium viscosity far less than fibrous lignocellulosic residues such as bagasse and contains very little lignin. While the *T. reesei* genome encodes a number of lignin degrading enzymes [34], it is generally not considered to significantly degrade lignin [35]. Lignin can also irreversibly bind cellulases [36] and thus leads to enzyme yield losses.

**Fig. 1:**
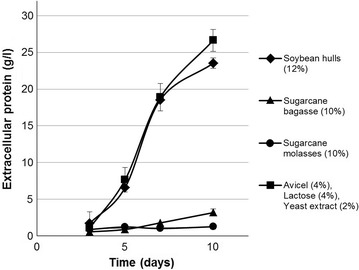
Extracellular protein production by *T. reesei* M44 in shake flask culture on select carbon sources. Extracellular protein concentrations measured from culture supernatant samples of *T. reesei* M44 cultivated on 12% soybean hulls, 10% sugarcane bagasse, 10% sugarcane molasses in mineral medium and a previously optimized combination of 4% Avicel, 4% lactose and 2% yeast extract in sugarcane vinasse

More crucially, our strain was found to secrete great quantities of extracellular protein when cultivated on milled soybean hulls. Figure 1 shows a comparison of cellulase secretion by *T. reesei* M44 on sugarcane molasses, sugarcane bagasse, soybean hulls, and cellulase inducer medium previously optimized for the strain. This inducer medium comprised Avicel microcrystalline cellulose, lactose, and yeast extract invinasse, the effluent water from sugarcane ethanol distillation. No enzymes were produced on sugarcane molasses, while only very minor amounts (3.2 g/l) were produced on sugarcane bagasse. Soybean hulls alone, however, induced the secretion of quantities of enzymes (23.5 g/l) approaching those obtained on the optimized inducer medium (26.6 g/l). None of the other residues evaluated induced production of more than 10 g/l of extracellular enzyme, leading us to focus our attention on soybean hulls.

**Fig. 2:**
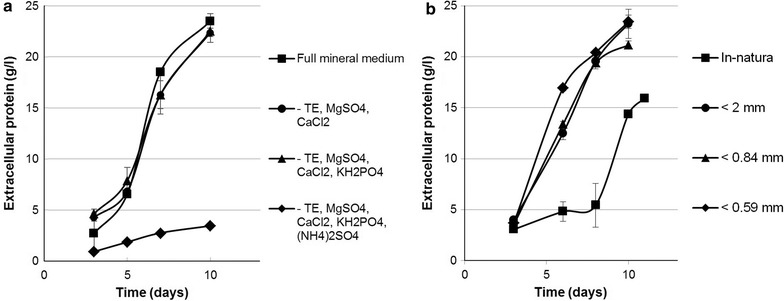
The effect of salt supplementation and substrate milling on extracellular protein production by *T. reesei* on soybean hulls. *T. reesei* (VTT-BR-C0019) was cultivated in shake flasks on soybean hull mineral medium under various conditions. **a** Extracellular protein production on 12% milled soybean hulls with full mineral medium, with the removal of trace elements, MgSO_4_ and CaCl_2_, with the removal of the aforementioned salts and KH_2_PO_4_ and in the absence of all added salts including (NH_4_)_2_SO_4_. Only the lack of (NH_4_)_2_SO_4_ had a clear negative impact on extracellular protein production. **b** Extracellular protein production on 12% *in*-*natura* soybean hulls or with the same amount of soybean hulls milled to pass a 2, 0.84 or 0.59 mm sieve

Soybean hulls were later found to provide nearly all necessary nutrients for the growth and production of enzymes in *T. reesei* cultures. By sequentially removing components of our mineral medium, we found that only the nitrogen source ammonium sulfate was not dispensable (Fig. 2a). However, ammonium sulfate is a relatively inexpensive salt, and liquid ammonia is routinely used to control pH in *T. reesei* fermentations [16, 20, 37, 38], thus directly providing for a nitrogen source. Additionally, we performed a simple evaluation in shake flasks on soybean hulls milled to different extents demonstrating that a <2 mm particle size was sufficient for achieving high enzyme titers (Fig. 2b). Extensive milling could significantly add to the cost of the use of this raw material. We were also able to achieve comparable titers (19.3 g/l) in bioreactors in a 96-h cultivation (Additional file 2: Figure S1), corresponding to an overall enzyme productivity of around 0.2 g/l h using only soybean hulls, and ammonium sulfate and ammonia as additional sources of nitrogen.

Although the results obtained with soybean hulls were promising, we estimated that the achieved titers and productivities would not suffice for industrial on-site cellulase production. Depending on the extent of milling, we found that soybean hulls could be used, at most, at concentrations ranging from 100 to 140 g/l without compromising medium aeration and cell growth in bioreactors, thus setting an upper limit for maximal obtainable enzyme titers. To achieve yet higher titers and productivities, a soluble carbon source would therefore be required.

### Creation of strain VTT-BR-C0019 secreting enzymes in the presence of repressing sugars

The most obvious choice of soluble carbon source in the context of a Brazilian sugarcane biorefinery was sugarcane molasses. Molasses is a relatively low-cost, high-density stream from sugar production that in addition to the sugars sucrose, glucose, and fructose contains several other nutrients, and is a common carbon source used for microbial fermentation. The very high sugar concentration of molasses (≥500 g/l) makes it ideal for use as a fermentation feed. However, the sugars present in molasses (sucrose, glucose, and fructose) are repressing sugars that would inhibit cellulase production in a conventional *T. reesei* strain. We therefore sought to modify our production strain to be able to use such sugars for cellulase production.

To broaden the range of carbon sources that could be used for cellulase production, we expressed a modified *xyr1* transcription factor under the constitutive pyruvate decarboxylase (*pdc1*) promoter [23] in our production strain. It has been shown that such a modification could lead to increased cellulase production and the expression of endoglucanase when *T. reesei* is cultivated on glucose [23]. We made a single amino acid substitution by replacing a valine residue at position 821 for a phenylalanine (V821F). Previous work suggested that a mutation in this region of the protein could further reduce the repression of enzyme secretion by glucose [25, 26].

**Fig. 3:**
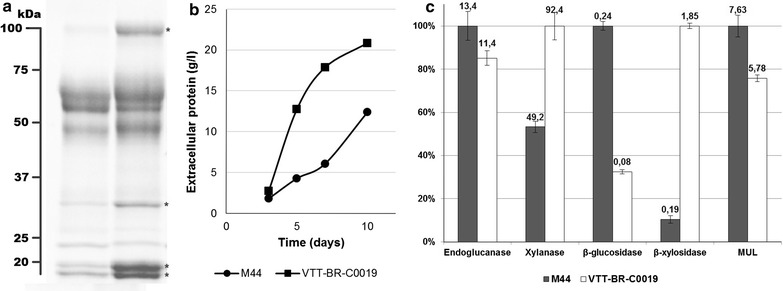
Production of enzymes by VTT-BR-C0019 in shake flasks on an inducing medium as compared to parental strain M44. *T. reesei* strains VTT-BR-C0019 and M44 were cultivated in shake flasks on an inducing medium comprising 4% milk whey, 4% Avicel and 1% yeast extract. **a** Culture supernatant samples from the last cultivation day (day 10) visualized on SDS-PAGE. *Left lane* Parental strain M44, Right lane: VTT-BR-C0019. *Asterisks* mark the clearly overexpressed proteins, likely corresponding to the main *T. reesei* xylanolytic enzymes. **b** Extracellular protein measured from cultivation samples of the parental strain M44 and VTT-BR-C0019. **c** Enzyme activity profile of the final cultivation day (day 10) samples. The* bars* represent the relative specific activities between the parental strain M44 and VTT-BR-C0019, while the numeric labels give the specific activities in units/milligram of protein

**Fig. 4:**
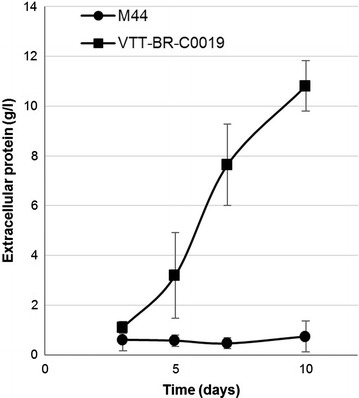
Production of extracellular protein by VTT-BR-C0019 in shake flasks on a repressing medium as compared to parental strain M44. *T. reesei* strains VTT-BR-C0019 and M44 were cultivated in shake flasks in mineral medium with 50 g/l glucose as carbon source and 10 g/l yeast extract as organic nitrogen source

The strain VTT-BR-C0019 was selected from shake flask screening of transformants. The strain demonstrated higher productivities and overall enzyme titers on an inducing mineral medium containing Avicel and milk whey (Fig. 3b), as well as an enrichment of the xylanolytic activities xylanase and β-xylosidase (Fig. 3c). Visualization of the secreted proteins by SDS-PAGE also showed clearly stronger bands, which based on their molecular weight likely correspond to the main xylanases (XYN1, XYN2, XYN3) and β-xylosidase (BXL1) of *T. reesei* (Fig. 3a). This alteration of enzyme profile could be advantageous in the hydrolysis of biomass, as conventional *T. reesei* enzyme preparations are normally considered to contain insufficient amounts of hemicellulolytic enzymes [11]. Most importantly, the strain was found to secrete significant amounts of enzymes also when cultivated on glucose (Fig. 4). Endoglucanase secretion on glucose by a *xyr1* overexpressing strain of *T. reesei* has previously been reported [23]. We also saw endoglucanase activity (14.5 U/mg), but additionally we observed significant activity toward xylan (170 U/mg) and 4-methylumbelliferyl-β-d-lactopyranoside (MUL—9.7 U/mg) in the enzymes produced on this carbon source. Cellobiohydrolase I (CBHI, Cel7A) is the primary *T. reesei* enzyme with activity toward MUL, but other *T. reesei* enzymes, particularly endoglucanase I (EGI, Cel7B) also exhibit some activity toward this substrate [37].

The secretome of VTT-BR-C0019 grown in a glucose medium as well as on other carbon sources continued to present the very low levels of β-glucosidase activity characteristic of *T. reesei* [39]. In fact, the overexpression of the modified XYR1_V821F transcription factor only seemed to further reduce the specific β-glucosidase activity of the enzymes produced by the strain (Fig. 3c). We therefore considered the overexpression of a β-glucosidase the most important second modification to the strain.

### Creation of strain VTT-BR-C0020 expressing beta-glucosidase from *Talaromyces emersonii*

**Fig. 5:**
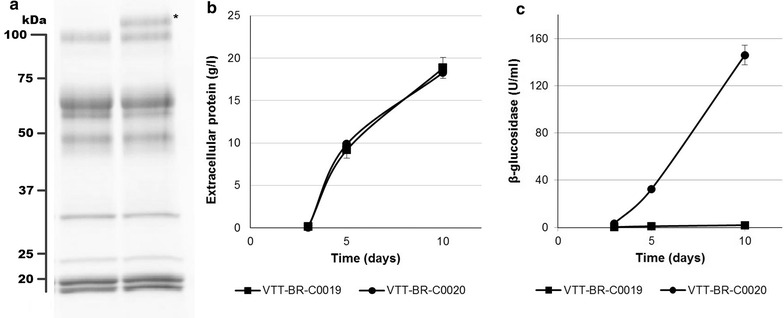
Production of enzymes by VTT-BR-C0020 in shake flasks on an inducing medium as compared to the parental strain VTT-BR-C0019. *T. reesei* strains VTT-BR-C0020 and VTT-BR-C0019 were cultivated in shake flasks on an inducing medium (1% yeast extract, 4% milk whey, 4% Avicel). **a** Culture supernatant samples from the last cultivation day (day 10) visualized on a SDS-PAGE gel. *Left lane* Parental strain VTT-BR-C0019, *right lane* VTT-BR-C0020. Additional band corresponding to the heterologous beta-glucosidase is marked with an *asterisk*. **b** Extracellular protein measured from cultivation samples of the parental strain VTT-BR-C0019 and VTT-BR-C0020. **c** β-Glucosidase activity measured from cultivation samples of the parental strain VTT-BR-C0019 and VTT-BR-C0020

To increase the β-glucosidase activity of the enzymes secreted by our strain, we overexpressed the Cel3A β-glucosidase from the moderately thermophilic fungus *Rasamsonia (Talaromyces) emersonii*. This enzyme has previously been expressed in *T. reesei* and characterized [30]. The constitutive expression of XYR1_V821F had led to a significant overexpression of xylanases (Fig. 3a, c), at levels we considered more than adequate for the hydrolysis of most types of pre-treated biomass. We therefore decided to utilize the *xyn1* promoter to drive the β-glucosidase expression. The use of more standard *T. reesei* cellulase promoters, such as that of cel7a, was avoided primarily for the fear of decreasing native cellulase expression. The *bar* gene conferring resistance to phosphinothricin (glufosinate) [40] was used as selectable marker. As previously, we selected a single transformant (VTT-BR-C0020) displaying the best characteristics (consistent high level of β-glucosidase production) in shake flask culture (Fig. 5). This strain was found to produce similar amounts of extracellular protein as the parental strain VTT-BR-C0019 on an inducing medium (Fig. 5b), but additionally up to 146 U/ml total or 8 U/mg specific β-glucosidase activity. The heterologous protein was also clearly visible on SDS-PAGE (Fig. 5a). We therefore had a strain secreting enzymes on repressing sugars and secreting higher levels of xylanolytic enzymes, as well as β-glucosidase, compared to the original strain M44.

### Evaluation of the enzymes produced using strain VTT-BR-C0020 on soybean hulls and sugarcane molasses

To evaluate the performance of the new strain VTT-BR-C0020, a bioreactor cultivation was performed with the batch medium comprising only soybean hulls and ammonium sulfate and with a feed comprising only sugarcane molasses. As the *T. reesei* genome encodes no invertase [41], the sucrose in the molasses was inverted using hydrochloric acid prior to its use as a feed. The fermentation was terminated after 120 h with the final extracellular enzyme titer reaching 30.8 g/l (Additional file 2: Figure S1), or about 50% more than what was achieved on soybean hulls alone. The overall enzyme productivity was also higher at about 0.26 g/l h.

**Fig. 6:**
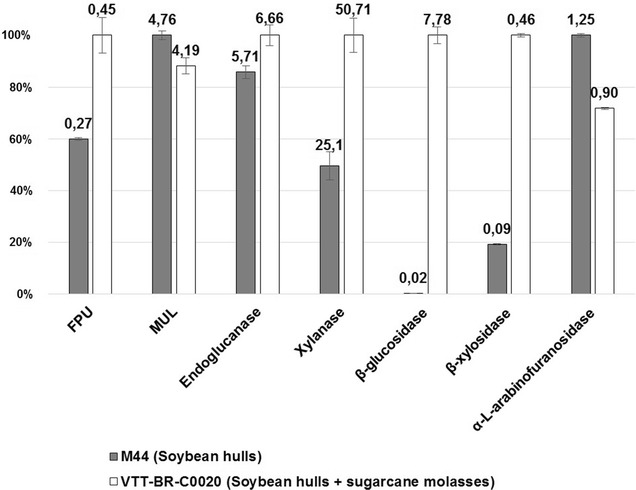
Profile of enzymes produced by original strain M44 on soybean hulls and strain VTT-BR-C0020 on soybean hulls and acid-inverted sugarcane molasses. Comparison of specific enzymatic activities quantified from culture supernatants of the original strain M44 cultivated on soybean hulls and the strain VTT-BR-C0020 cultivated on soybean hulls and acid-inverted sugarcane molasses. The bar heights give relative specific activities between the two enzyme mixtures, while the numeric labels give the specific activities in units/milligram of protein

To understand the potential of the produced enzyme mixture, we used it to hydrolyze industrial hydrothermally pre-treated sugarcane straw. As the original strain M44 produces no enzymes on molasses (Fig. 1), the performance of the new enzyme was compared to the culture supernatant of the original strain cultivated on soybean hulls alone (Additional file 2: Figure S1). Figure 6 shows the relative specific activities measured from these two enzyme preparations. As expected, the principal differences were to be found in the activities xylanase, β-xylosidase, and β-glucosidase. The enzymes produced by VTT-BR-C0020 showed an approximately twofold, fivefold and 400-fold increase in these activities, respectively. Activities reflecting some of the main cellulases (endoglucanase and MUL) remained relatively unaltered.

**Fig. 7:**
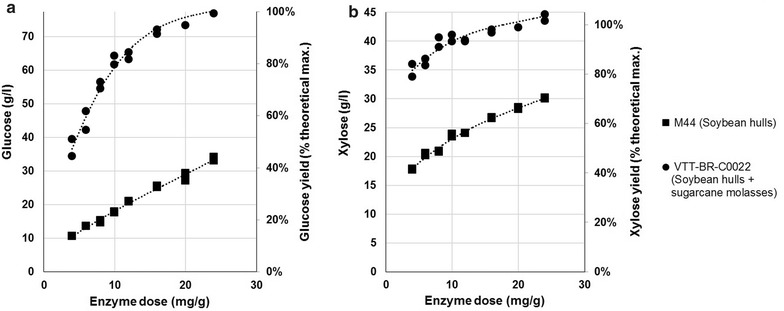
Relative performance of the enzymes produced by the original strain M44 and the strain VTT-BR-C0020 in the hydrolysis of industrial hydrothermally pre-treated sugarcane straw. The ability of the enzymes produced by M44 on soybean hulls to hydrolyze industrial hydrothermally pre-treated sugarcane straw was compared to the enzymes produced by VTT-BR-C0020 on soybean hulls and sugarcane molasses. The used enzyme dose varied from 4 to 24 mg/g substrate dry matter, and the reactions were carried out for 72 h at 45 °C at a total substrate solids loading of 20%. **a** Glucose liberation as measured by HPLC, **b** xylose liberation as measured by HPLC. Results displayed as concentration (g/l—primary axes) and as a percentage of the theoretical maximum (%—secondary axes)

The hydrolysis results show a dramatic increase in the ability of the improved enzymes to release glucose (Fig. 7a) and xylose (Fig. 7b) from the pre-treated sugarcane straw. Total monomeric sugar concentrations (glucose + xylose) in the hydrolysate surpassed 100 g/l with an enzyme dose of 10 mg/g total substrate solids. The result is probably the consequence of the increased β-glucosidase activity and the additional xylanolytic activities, particularly β-xylosidase.

### Creation of strain VTT-BR-C0022 expressing invertase from *Aspergillus niger*

The previous results showed that it would be possible to produce a well-performing enzyme using the strain VTT-BR-C0020 and only the low-cost raw materials soybean hulls, sugarcane molasses, and (NH_4_)_2_SO_4_. However, as *T. reesei* lacks a native invertase [41], an additional process step was required to hydrolyze the sucrose in molasses with acid. Aside from adding to process complexity, such a step might generate compounds inhibitory to the enzyme-producing fungus. Indeed, our method of acid inversion was found to lead to a significant decrease in tolerance by our *T. reesei* strain toward this carbon source. In shake flask culture, the strain VTT-BR-C0020 only tolerated the acid-inverted molasses up to a total reducing sugar (TRS) concentration of about 50 g/l, while *in*-*natura* molasses was tolerated up to 200 g/l TRS, the highest concentration evaluated (data not shown). To reach yet higher enzyme concentrations, more concentrated soluble carbon source would be required. A strain able to consume sucrose was therefore obviously desirable.

*Trichoderma reesei* has previously been engineered to express the suc1 invertase from *Aspergillus niger* [41]. We used the same gene, including the native *A. niger* promoter, to transform VTT-BR-C0020 with the aim of providing low-level invertase expression sufficient for growth on sucrose. Invertase would not contribute to biomass hydrolysis, so high-level expression was undesirable. This gene was transformed together with an overexpression construct for the activator of cellulase expression *ace2* [42], driven by the pdc1 promoter. The gene *thi4* [43] conferring resistance to pyrithiamine was used as a selectable marker (see Additional file 3: Table S2 for vector design). Transformants were screened in deep well plates containing mineral medium with sucrose as the only carbon source. The transformant exhibiting the best growth on sucrose mineral medium (VTT-BR-C0022) was selected as our final strain.

**Fig. 8:**
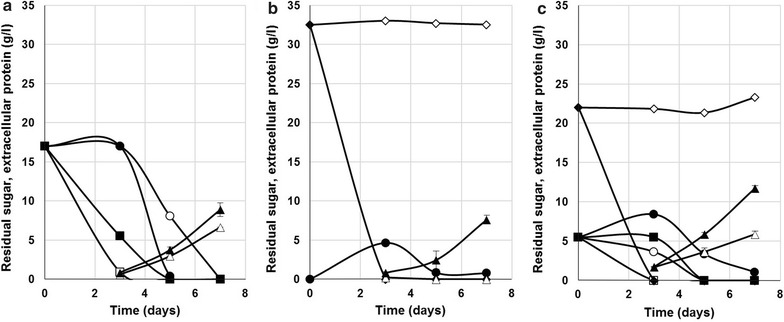
Utilization of sugars and production of extracellular proteins by VTT-BR-C0022 as compared to parental strain VTT-BR-C0020. Consumption of glucose, fructose and sucrose, and secretion of extracellular protein by invertase-expressing strain VTT-BR-C0022 and parental strain VTT-BR-C0020 cultivated on three different carbon sources: **a** pure glucose + fructose, **b** pure sucrose and **c** sugarcane molasses. In all cases the carbon source was used at a concentration of 30 g/l and supplemented with 3 g/l yeast extract as an organic nitrogen source

To study the behavior of the final strain, we performed shake flask cultivations comparing it to the parental strain VTT-BR-C0020 using three different carbon sources: (a) a combination of pure glucose and fructose, (b) pure sucrose, and (c) sugarcane molasses. Figure 8 shows the consumption of sugars and production of extracellular protein by the two strains. On glucose and fructose, strain VTT-BR-C0022 appeared to produce slightly higher concentrations (8.6 g/l) of enzymes as compared to the parental strain (6.9 g/l). Production of enzymes by VTT-BR-C0022 on pure sucrose reached similar levels (7.9 g/l) to those observed on the mixture of glucose and fructose, while as expected, no sucrose was consumed and no enzymes produced by the parental VTT-BR-C0020. Molasses seemed to be a better carbon source than pure sugars, with VTT-BR-C0022 reaching final enzyme titers of 11.8 g/l compared to 8–9 g/l for pure sugars. The parental strain again consumed no sucrose, but was able to produce up to 5.9 g/l of enzyme on the glucose and fructose contained in molasses alone.

**Fig. 9:**
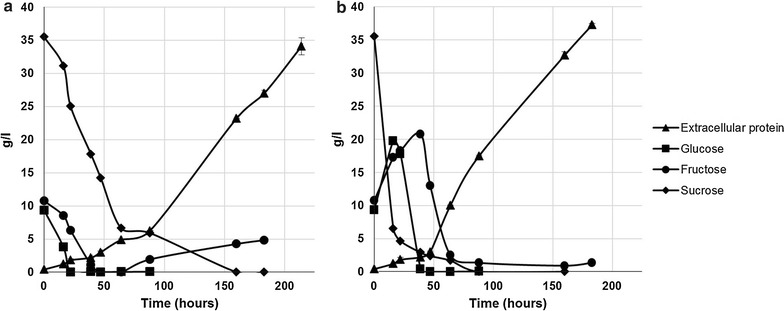
Sugar consumption and extracellular enzyme production by strain VTT-BR-C0022 in sugarcane molasses -containing media in bioreactor cultivation. Strain VTT-BR-C0022 was cultivated in bioreactors on sugarcane molasses based media. Residual glucose, fructose and sucrose, and extracellular protein measured from cultivation supernatant samples. **a** Cultivation with only 50 g/l total sugars from sugarcane molasses in batch phase and a sugarcane molasses feed. **b** Cultivation with 50 g/l total sugars from sugarcane molasses supplemented with 50 g/l milled soybean hulls in batch phase and a sugarcane molasses feed

To study the sucrose consumption and enzyme production of the final strain VTT-BR-C0022, we also performed fermentations with non-inverted sugarcane molasses (Fig. 9). In one reactor, the inoculum and batch-phase growth were carried out on sugarcane molasses only (Fig. 9a), while in the other one, the inoculum and batch medium were supplemented with 50 g/l of milled soybean hulls (Fig. 9b). In both cases, the strain VTT-BR-C0022 was able to invert all sucrose in the batch medium and in the feed. Only slight accumulation of fructose was observed in the pure molasses cultivation. In these cultivations, we were able to reach extracellular protein concentrations of 34.1 g/l in 214 h (0.16 g/l h) on molasses alone (Fig. 9a), and 37.3 g/l in 183 h (0.20 g/l h) when combined with soybean hulls (Fig. 9b). These results demonstrate that using the described strain and raw materials, industrially relevant enzyme productivities and titers could be achieved.

### Use of whole enzyme broth for hydrolysis and SSF

One advantage of producing cellulases at their final site of use is considered to be the possibility of avoiding clarification of the enzyme. However, only a limited number of studies have been performed using a whole enzyme broth, including fungal mycelia, to hydrolyze lignocellulosic biomass [7, 8]. Additionally, to the best of our knowledge, the possible effects of fungal mycelia on the ethanologen have not been studied in detail. To understand if whole enzyme broth produced using our method could indeed be used for hydrolysis and ethanol fermentation, we performed simultaneous saccharification and fermentation (SSF)-type reactions in shake flasks. We used aseptically taken final samples of the fermentation shown in Fig. 9b to hydrolyze industrially pre-treated sugarcane straw (GranBio Ltda.).

**Fig. 10:**
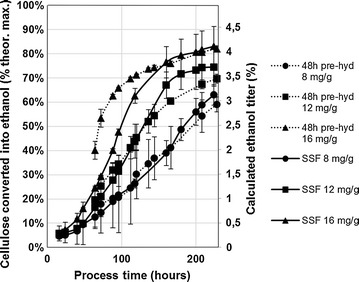
Hydrolysis and fermentation of washed industrial sugarcane straw using whole enzyme broth including fungal mycelia and other solids. Whole enzyme broth containing fungal mycelia and other solid residues was recovered from the final sample of the bioreactor cultivation of strain VTT-BR-C0022 on soybean hulls and sugarcane molasses (Fig. 9b). The whole enzyme broth was dosed in hydrolysis at 8, 12, and 16 mg/g. The reactions were carried out in two modes: with a 48-h pre-hydrolysis step at 45 °C followed by yeast addition and fermentation at 33 °C and pure simultaneous saccharification and fermentation carried out at 33 °C. The *X-axis* considers the time of both the hydrolysis and fermentation steps. The bottles were weighed periodically, and the loss of weight due to evolution of CO_2_ was converted into values of cellulose to ethanol conversion (primary *Y-axis*) and ethanol titer (secondary *Y-axis*)

For the SSF experiments, it was necessary to wash the pre-treated substrate to allow growth and ethanol production by *S. cerevisiae*. The experiments were carried out with a 48-h pre-hydrolysis step at 45 °C prior to fermentation at 33 °C, or as pure SSF reactions at 33 °C. It was known from experience that 45 °C was sufficient to inactivate *T. reesei* cells (although not spores). Similar final ethanol titers were achieved in both cases, suggesting that the presence of viable fungal cells at the beginning of fermentation did not significantly affect ethanol yield (Fig. 10). Ethanol yields ranging from 60 to 80% of the theoretical maximum were achieved with the different enzyme doses.

## Discussion

Enzymes remain a major cost factor for the nascent lignocellulosic fuels and chemicals industry. The operational cost of enzymes arises from two main components: the quality of the enzyme (i.e., how much enzyme is needed to hydrolyze a given quantity of biomass) and the cost of each kilogram of enzyme to the end user. The quality of cellulase enzymes and the factors contributing to it are often exhaustively discussed. However, less attention is generally given to the cost of enzyme ($/kg) and the factors contributing to this cost, and very little information is available on the production costs of commercial cellulases [1].

In one of the most thorough analyses on the topic to date, with the stated objective of adding transparency to the enzyme cost discussion, Humbird et al. modeled a cellulase production process co-located at a corn ethanol mill [5]. This techno-economic model assumed the use of glucose ($580/t) as carbon source and found that it accounted for over 50% of the total cost of the produced enzyme ($4.24/kg). Other major contributors were the capital invested in equipment (21%) and the electricity used to run the process (13%). Even so, the modeled process provided for better economics than the commercially delivered enzymes at the time.

However, this theoretical model assumed a highly performing cellulase production process with an enzyme productivity of 0.42 g/l h and final titer of 50 g/l using only glucose as carbon source. In addition, the produced enzyme was assumed to be relatively well performing and dosed at 20 mg/g of glucan (corresponding roughly to 10 mg/g dry substrate, the metric used in this study). To the best of our knowledge, these figures are beyond what is reported in the literature, suggesting that sufficiently high-performing strains and processes are not available in the public domain. Previous reports detail hypercellulolytic strains of *T. reesei* producing up to 40 g/l of total extracellular protein in 8–10 days (≤0.2 g/l h) on media containing inducers (lactose or cellobiose) [16, 44].

As a means of decreasing enzyme cost, many studies cite the use of lignocellulosic biomass as a carbon source for enzyme production [1, 4, 6, 9–12], and the concept does seem to offer many benefits. By definition, the lignocellulosic biomass used for ethanol production should be of low cost, and the use of the same biomass could allow the enzyme-producing fungus to produce a more specific enzyme mixture for the hydrolysis of the biomass. We initially also considered this alternative. However, the limitations of using lignocellulosic biomass for high-yield enzyme production rapidly became apparent. The principal drawbacks are related to viscosity, nutrient availability, and toxicity.

Fibrous biomass residues have a tendency to absorb great quantities of water, even when milled, and thus lead to highly viscous media. Through our experience, we expect that *in*-*natura* sugarcane biomass residues could at most be used at concentrations of around 50 g/l in large bioreactors, setting a very low upper limit for achievable enzyme titers. In addition, *in*-*natura* biomass is recalcitrant and contains a relatively high proportion of lignin, which soft-rot fungi do not readily consume. Lignin can also non-productively and irreversibly bind cellulases [36], thus potentially decreasing yield. We assume that all these factors played a role in the low extracellular enzyme titers achieved with our strain on *in*-*natura* sugarcane straw (Fig. 1).

Pre-treated biomass is less recalcitrant, but contains an even higher proportion of lignin, aside from toxic inhibitors such as furfural, hydroxymethylfurfural (HMF), and phenolic compounds. In our experience, hydrothermally pre-treated biomass is toxic to *T. reesei* even at very low concentrations (<30 g/l). Low cellulase yields and productivities mean prohibitive economics from capital expenditure, even if the raw material cost was next to zero.

Another techno-economic model illustrates some of these points [1]. This model considered inexpensive steam-exploded poplar ($60/t) as carbon source at high consistency (~30% solids) in *T. reesei* -fermentation for enzyme production. Although such a process in our experience seems impossible in practice due to the questions of toxicity and viscosity, the model nonetheless arrived at an enzyme price of over $10/kg. Notably, costs associated with the financing and operation of equipment accounted for 65% of the total cost. The financing for and operation of equipment required for submerged, aseptic, aerobic fermentation are costly, and therefore the productivity of the equipment is paramount for the overall economics of the process.

Due to these considerations, we excluded lignocellulosic biomass as an alternative and expanded our scope to identify alternative low-cost Brazilian residues that would serve better for the purpose. Of the options considered, soybean hulls presented by far the most attractive characteristics. Soybean hulls have been used for cellulase production using *T. reesei* in at least two prior studies [45, 46], while the more expensive soy derivative soy bran has been used in other publications [47, 48]. We were able to reach productivities of 0.2 g/l h and titers of 20 g/l using this residue alone (Fig. 1; Additional file 2: Figure S1). After modification of our production strain, we included sugarcane molasses, a relatively inexpensive, high-density sugar stream as an additional carbon source, increasing the productivity (0.26 g/l h) and titer (30 g/l). These figures reach more than 50% of those assumed in the techno-economic model of Humbird et al. [5], and the productivity is similar to that assumed by Klein-Marcuschamer et al. [1].

We estimated that the nutrients in the medium containing soybean hulls ($100/t), sugarcane molasses ($205/t total reducing sugar—TRS),and ammonium sulfate ($331/t) used to produce enzymes with VTT-BR-C0020 (Additional file 2: Figure S1) to have cost around 25 $/m^3^ (using the average exchange rate for 2015 of $1 = R$ 3.467). Considering a final enzyme titer of 30 kg/m^3^, this would signify a growth medium cost contribution of less than $1/kg of the produced enzyme. Factoring in other operational costs and capital cost, the final price of the enzyme produced with these raw materials could by estimate be as low as $2–4/kg. Our process therefore provides an intermediate between the previously discussed high carbon source price/low capital cost model [5], and the low carbon source cost/high capital cost model [1]. Of note is that both models also included additional nutrient sources, such as corn steep liquor and salts, adding to nutrient cost. Table 1 presents a comparison between some of the key metrics of the discussed techno-economic models and the experimental data presented here.

**Table 1:** Comparison of some of the key metrics of the enzyme production processes described in the techno-economic models of Humbird et al. [5] and Klein-Marcuschamer et al. [1], and the data from this study

	Humbird [5]	Klein-Marcuschamer [1]	This study^a^
Carbon source	Glucose	Steam-exploded poplar	83% Soybean hulls, 17% sugarcane molasses
C-source price ($/t)	580	60^b^	120
Enzyme yield from C-source (g/g)	0.24	0.13^c^	0.21
Final titer (g/l)	50	46	30.6
Production time (h)	120	192	120
Productivity (g/l h)	0.42	0.24	0.26
Nutrient cost ($/kg enzyme)	2.63	2.84	~0.85
Total enzyme cost ($/kg)	4.24	10.14	N/D

^a^Considering the fermentation performed with VTT-BR-C0020 on a medium with a final concentration of 125 g/l soybean hulls and 25 g/l molasses TRS (Additional file 1: Figure S1)

^b^Cost assumed in the base scenario for native poplar, not including the cost of pre-treatment

^c^Yield from pre-treated poplar total solids

However, in-depth techno-economic modelling would be required to assess the true cost of the enzyme produced using the process described in this study. One particular consideration is the effect on the process costs of using high solids in enzyme production. On the other hand, we were able to show that high enzyme titers are also achievable without the addition of soybean hull solids using the invertase-expressing strain VTT-BR-C0022 (Fig. 9b).

Besides enzyme cost, we also improved the quality of the enzymes secreted by our production strain by overexpressing the native xylanases of *T. reesei* (through the overexpression of a mutated *xyr1*) and adding a heterologous β-glucosidase from *T. emersonii*. These improvements allowed the enzyme to be used in hydrolysis at enzyme loadings similar to those assumed by Humbird et al. [5] and Klein-Marcuschamer [1] (20 mg/g glucan). Using the roughly corresponding dose of 10 mg/g dry matter, our enzyme hydrolyzed over 80% of the substrate cellulose and over 90% of the substrate xylan in 72 h, which should be sufficient for an industrial cellulosic ethanol process. Strikingly, similar glucose yields were achieved with a more than 80% reduction in enzyme loading compared to the enzyme produced by the original strain (Fig. 7), emphasizing the importance of the modifications made.

In the present study, we also showed that whole fungal fermentation broth containing *T. reesei* mycelia and other solid residues could be used for biomass hydrolysis without any prior processing (Fig. 10), which could potentially bring large savings. The overall cost contribution of enzymes to a cellulosic ethanol process needs to be evaluated on a case-by-case basis.

We also note that this study relied only on a small number of genetic modifications and we assume that significant improvements could be achieved with further strain development. Additional transcription factor modifications could significantly improve enzyme yields and production kinetics. For example, the downregulation of ace1 has been shown to increase enzyme yields in *T. reesei* Rut-C30 [23], and novel cellulase activators have recently been described [49]. Optimization of the fermentative process might also result in productivity gains. In addition, expressing other enzymes could have a further beneficial impact on enzyme performance. In particular, the addition of lytic polysaccharide monooxygenases (LMPOs) could be expected to improve the hydrolysis performance of the enzymes secreted by the strain [50].

## Conclusions

The aim of the present study was to develop a simple and cost-effective process for the production of cellulases that could be implanted in the context of a Brazilian sugarcane mill. We developed a low-cost growth medium based on soybean hulls and sugarcane molasses and engineered our *T. reesei* production strain for higher productivity and better enzyme profile using a number of previously described modifications. With further improvements, such a system could allow the local production of low-cost cellulase in biomass rich regions where limiting infrastructure or other factors favor a distributed enzyme production model.

## Methods

### Strains and propagation

The strain *T. reesei* M44 and its derivatives were routinely cultivated on petri dishes containing Potato Dextrose Agar (Difco). After 6–10 days, spores were collected in spore solution (20% glycerol, 0.8% NaCl, 0.025% Tween 20), filtered through sterile cotton, quantified using a hemocytometer and frozen at −80 °C for long-term storage.

### Shake flask and microplate cultivations

The *T. reesei* strains were routinely cultivated in 500 ml baffled Erlenmeyer flasks by inoculating 2*10^7^ spores in 100 ml of mineral medium containing 10 g/l (NH_4_)_2_SO_4_, 15 g/l KH_2_PO_4_, 0.59 g/l MgSO_4_, 0.45 g/l CaCl_2_, 5 mg/l FeSO_4_·7H_2_O, 2 mg/l CoCl_2_·6H_2_O, 1.6 mg/l MnSO_4_·4H_2_O, 1.4 mg/l ZnSO_4_·7H_2_0 and 100 mM buffer (PIPPS-Calbiochem) with the initial pH adjusted to 4.8 with KOH. Solid carbon sources were autoclaved together with the media within the flasks. When milled solid substrates were used, these were prepared using a Wiley knife mill and milled to pass a 0.59 mm screen unless otherwise indicated. Sugarcane molasses were autoclaved separately, while pure soluble carbon sources were filter sterilized using a 0.22-μ filter, before addition to the sterilized base media. To invert the sucrose in molasses, a sample was first diluted with water to a total sugar concentration of around 500 g/l, the pH was lowered to 2.0 with hydrochloric acid, and the sample then autoclaved using a standard liquid cycle. This procedure was found to invert more than 95% of the sucrose contained in molasses. pH was then corrected to 4.0 with 10 M KOH. Cultivations were carried out in a shaking incubator (Infors HT Multitron) with 200 rpm at 28 °C. Samples were withdrawn aseptically on cultivation days 3, 5, 7, and 10, centrifuged at 14,000*g*, and the supernatants were stored at −20 °C for analysis. In some cases, screening of transformant clones was performed in 24 deep well microplates sealed with Breath-Easy membranes (Sigma), with each well containing 6 ml of media of the same basic composition. Used carbon sources and deviations from the basic media composition are detailed in “Results” section.

### Evaluation of low-cost residues

Several industrial residues were evaluated for their potential as carbon sources for cellulase production. Samples of were sourced from local Brazilian producers, in some cases milled and then evaluated in shake flask cultures using the previously described mineral medium supplemented with the given residues at concentrations varying from 30 to 120 g/l. Materials were considered toxic to *T. reesei* if no germination of spores was observed by 5 days of shake flask cultivation at the lowest evaluated residue concentration (30 g/l). Rheology was evaluated visually by observing the amount of free liquid in the medium and the ease of agitation at different residue concentrations. Availability and price estimates were gathered from several official Brazilian sources, including Companhia Nacional de Abastecimento (CONAB), Ministério de Agricultura, Pecuária e Abastecimento (MAPA), Instituto Brasileiro de Geografia e Estatística (IBGE) and Indústria Brasileira de Árvores (IBÁ). The opportunity cost for a sugarcane mill of utilizing molasses for enzyme production (R$ 712/t TRS) was a personal industry communication. The availability of a residue for cellulase production was considered with regard to both total annual production, as well as seasonal and regional variability. The ability to induce enzyme production was evaluated based on the highest extracellular protein titer observed in shake flask cultures.

### Fermentations

Fermentations were performed using the BioFlo/CelliGen 115 system (Eppendorf) and water-jacketed 3.0-l vessels. As milled soybean hulls and sugarcane molasses were found to provide most nutrients required by *T. reesei*, the standard fermentation medium comprised only 2% (NH_4_)_2_SO_4_ and 1 ml/l of J647 antifoam (Struktol) in addition to the described carbon sources. Aeration was maintained at 0.7 VVM compressed air, pH between 4.0 and 5.0 using 2 M phosphoric acid and 15% ammonia, and DO above 20% with an agitation cascade (400–1000 rpm). The used initial volume was 1–1.2 L, and the reactors were inoculated with 1:10 volume of 3–7 day old shake flask preculture of the same media composition as the fermentation batch medium. Samples were withdrawn at regular intervals, centrifuged at 21,000*g* for 5 min and the supernatants stored at −20 °C for analysis. Final fermentation samples were withdrawn for hydrolysis experiments. Whole broth samples were used directly, while separate samples were clarified by first centrifuging at 9000*g*, filtering through glass fiber filters (Whatman GF/B) and finally through 0.45 PVDF membranes before storage at −20 °C. When molasses was used as feed, the feeding was terminated 12–24 h prior to terminating the cultivation. Fermentations were terminated once pH rose markedly above the set point (4.0), and DO showed a continuous upward trend for more than 6 h, which was interpreted as an indication of starvation.

In the fermentation with VTT-BR-C0020 (Additional file 2: Figure S1), the inoculum and batch medium contained 130 g/l milled soybean hulls, and acid-inverted molasses with a concentration of total sugars of 340 g/l were fed from 24 to 96 h of fermentation at an average rate of 0.38 g/l h total sugars.

In the fermentations using strain VTT-BR-C0022 with only molasses (Fig. 9a), the feed was maintained from 65 to 195 h of cultivation at a rate of 1 g/l h total sugar. In the cultivation supplemented with soybean hulls (Fig. 9b), the feed was maintained from 72 to 168 h of cultivation at a rate of 0.5 g/l h total sugar.

### Molecular cloning

All restriction enzymes used in this study were FastDigest enzymes from Thermo Scientific, except for BsaI-HF (NEB). The DNA polymerase (Phusion), ligase (T4 ligase), and DNA ladders were also from Thermo Scientific. The used cloning host (*Escherichia coli* DH5a) as well as the kits used for purifying DNA from *E. coli*, fungi, and agarose gels were from Zymo Research. Sequencing of plasmids was performed by Helixxa Ltda. using an Applied Biosystems 3500 capillary electrophoresis system.

The plasmids used for the transformation of *T. reesei* were constructed using the type-II restriction endonuclease *Bsa*I. The plasmids were assembled from 5–6 individual linear fragments with compatible 4-bp 3′-overhangs resulting from digestion with BsaI (Additional file 3). Unlike in the GoldenGate method [51], we performed the digestion and subsequent ligation reactions separately. Each fragment comprised one functional unit of the construct, either the promoter or gene of interest or terminator or marker or the vector backbone (derived from pUC57-Kan), and in selected cases, we added an additional sixth fragment. The individual linear fragments used in ligation were derived directly from PCR-amplification or released from specific pUC57-based storage plasmids. The PCR products or plasmids were digested using BsaI-HF (NEB), the desired DNA-fragment purified from an agarose gel, the DNA was eluted in deionized water and quantified using Infinite 200 NanoQuant (Tecan), and the sample was stored at −20 °C until use in ligation. The ligation reactions were carried out for a minimum of 3 h at room temperature according to the enzyme manufacturer’s protocol and 5 µl of the final product used to transform chemically competent Zymo 5a cells (Zymo Research).

The plasmid pVTTBR43 was constructed by ligating the fragments *pdc* promoter, *xyr1_V821F*, *pdc* terminator and *hph*-*amdS* cassette into the pUC57-Kan derived backbone.

The plasmid pVTTBR54 was constructed by ligating the fragments *xyn1* promoter, *TeCel3A*, *xyn1* terminator, *bar* cassette, CBHI 3′-flank into the pUC57-Kan derived backbone.

The plasmid pVTTBR80 was constructed by ligating the fragments *pdc* promoter, *ace2*, *pdc* terminator, *thi4*, CBHI 3′-prime flank into the pUC57-Kan derived backbone. InFusion cloning (Clontech) was then used to replace the CBHI 3′-flank fragment with the *suc1* (*sucA*) gene of *Aspergillus niger* to create the plasmid pVTTBR92.

More details on vector construction and the origin of each linear fragment are provided in Additional file 3 and used primers are listed in Additional file 4.

### Creation of *T. reesei* strains VTT-BR-C0019, -C0020, and -C0022

Transformation of *T. reesei* was performed essentially as described in [52]. In each case, 5–10 µg of plasmid was digested with suitable FastDigest (Thermo Scientific) enzymes and the product run on a 0.8% agarose gel. The desired linear fragment (the transformation cassette) was extracted from the gel and 2–5 µg of DNA used to transform *T. reesei* protoplasts, which were then plated in selective top agar. The selection plates comprised 10 g/l (NH_4_)_2_SO_4_, 15 g/l KH_2_PO_4_, 0.59 g/l MgSO_4_, 0.45 g/l CaCl_2_, 5 mg/l FeSO_4_·7H_2_O, 2 mg/l CoCl_2_·6H_2_O, 1.6 mg/l MnSO_4_·4H_2_O, 1.4 mg/l ZnSO_4_·7H_2_0, 15 g/l agar and 10 g/l glucose as carbon source. Colonies emerging from the top-agar were picked between 5 and 10 days after plating and re-streaked on selective plates. After 5–7 days, a spore suspension was prepared from these plates, diluted sufficiently and plated on selective plates containing 0.1% Triton X-100 as a colony restrictor. Isolated colonies arising from single spores were then picked and streaked on PDA to generate a final suspension of spores that could be used for cultivations in liquid media. Cultivations in liquid media were used to verify desired phenotypes and prepare cell mass for genomic DNA extraction using the ZR Fungal/Bacterial DNA MiniPrep kit (Zymo Research). Integration of the transformation cassette was verified from genomic DNA using PCR and suitable primer combinations.

To create strain VTT-BR-C0019, protoplasts prepared from M44 were transformed with the linear fragment resulting from the digestion of pVTTBR43 with *Mss*I. The protoplasts were plated in top-agar containing 50 µg/ml Hygromycin B (Calbiochem). 5 purified transcription factor transformants were screened in shake flask culture for their ability to secrete more extracellular enzymes than the parental strain, and the best transformant (VTT-BR-C0019) was selected for further improvement.

To create strain VTT-BR-C0020, protoplasts prepared from VTT-BR-C0019 were transformed with the linear fragment resulting from the digestion of pVTTBR54 with *Mss*I and *Nhe*I. The protoplasts were plated in top-agar containing 1 mg/ml glufosinate-ammonium (Sigma). 9 purified clones were screened in shake flask culture for their ability to secrete more beta-glucosidase (pNPGase) than the parental strain, and the best one (VTT-BR-C0020) was selected for further improvement.

To create strain VTT-BR-C0022, protoplasts prepared from VTT-BR-C0020 were transformed with the linear fragment resulting from the digestion of pVTTBR92 with MssI. The protoplasts were plated in top-agarose containing 100 µg/ml pyrithiamine (Sigma) and sucrose instead of glucose as the carbon source. 10 purified clones were screened in microplate culture for their ability to produce invertase and grow on mineral medium with sucrose as the only carbon source, and the best one (VTT-BR-C0022) was selected as the final strain.

### Enzyme sample analysis

Cultivation supernatant samples from shake flasks and bioreactors were routinely analyzed for total protein content and enzymatic activities.

For quantifying protein, the sample was first diluted to a final concentration of 0.3–1.5 g/l in 50 mM pH 5.0 Na-citrate buffer. A 200 µl sample was combined with 800 µl ice-cold acetone in a 2 ml Eppendorf tube, mixed by inverting the tube several times and then maintained at −20 °C for 1 h. The precipitated proteins were pelleted by centrifuging at 14,000*g* and 4 °C for 5 min. The supernatant was removed and the pellet was air-dried for 5 min before resuspending in the original volume (200 µl) of buffer. The protein concentration was then quantified using the DC protein kit (BioRad) based on the method of Lowry [53] using Bovine Serum Albumin (BSA) as standard. Filter paper activity (FPase) was measured using the standard method [54].

The enzymatic activities β-glucosidase, β-xylosidase and α-l-arabinofuranosidase were measured using the substrates 4-nitrophenyl-β-d-glucopyranoside (pNPG), 4-nitrophenyl-β-d-xylopyranoside (pNPX) and 4-nitrophenyl-α-l-arabinofuranoside (pNPA), respectively. The reactions were carried out in 50 mM citrate buffer at pH 5.0 and at 50 °C for 10 min, the reactions terminated by adding one volume of 1 M NaCO_3_ and the released 4-nitrophenol quantified by measuring absorbance at 405 nm.

MUL activity was measured using the substrate methylumberriferyl-β-d-lactoside [37]. The primary *T. reesei* enzymes active toward this substrate are the GH7-family proteins cellobiohydrolase I (CBHI, Cel7A) and endoglucanase I (EGI, Cel7B), while other enzymes might also display limited activity. The reactions were carried out in 50 mM citrate buffer at pH 5.0 and 50 °C for 10 min, the reactions were terminated by adding one volume of 1 M NaCO_3_ and the released methylumberriferone quantified by measuring fluorescence at 445 nm using an excitation wavelength of 380 nm.

Endoglucanase and xylanase activities were measured using the substrates carboxymethylcellulose (CMC) and beechwood xylan, respectively. The reactions were carried out in 50 mM citrate buffer at pH 5.0 and at 50 °C for 5 or 10 min, the reactions were terminated by adding 1.5 volumes of DNS reagent. The reactions were heated for 5 min at 95 °C, and the released reducing sugars were quantified by measuring absorbance at 540 nm. Standard curves were prepared using pure glucose or xylose as appropriate.

Unless otherwise noted, error bars represent standard deviation from biological and experimental duplicates. In all cases, one unit of enzymatic activity was defined as the amount of enzyme releasing one micromole of product from the substrate in one minute under the reaction conditions specified. All substrates and standards used in the enzyme assays were from Sigma.

For visualization of enzyme samples on polyacrylamide gels, 15–20 µg samples based on the Bio-Rad DC measurement were loaded into individual wells on 4–20% Criterion TGX Strain-Free precast gels (Bio-Rad), run for 30 min with 200 V and gel images were captured using the Gel Doc EZ system (Bio-Rad).

### Hydrolysis

Hydrolysis reactions were performed in miniature scale using the Intellimixer agitation device (Elmi) set to program 2u18 and maintained at a temperature of 45 °C in an incubator cabinet. The substrate used was hydrothermally pre-treated sugarcane straw from an industrial facility (GranBio, São Miguel dos Campos, AL, BR), a kind gift of CTO Gonçalo Pereira. The substrate pH was adjusted to 5.0 using 10 M NaOH. Each individual hydrolysis reaction was set up to have a total mass of 1 g within a 2 ml Eppendorf tube. 200 mg of substrate (dry-basis) was added into each tube. The reaction was then completed to 1 g by adding distilled water, enzyme, NaN_3_ as an anti-microbial agent to a final concentration of 0.02% and pH 5.0 citrate buffer to a final concentration of 50 mM. After 72 h the entire contents of each tube was recovered in 9 ml of deionized water in a 15 ml Falcon tube, mixed thoroughly and centrifuged for 10 min at 2880*g*. A sample (1 ml) of the supernatant was transferred to a 1.5 ml Eppendorf tube, boiled for 10 min and then stored for analysis. Total reducing sugar was quantified using the DNS method and glucose as standard, and glucose and xylose were quantified using HPLC. To translate the results into degree of hydrolysis, samples of the pre-treated material were hydrolyzed with sulfuric acid according to the NREL protocol TP-510-42618 to quantify the maximal potential glucose and xylose.

### Shake flask hydrolysis and fermentation experiments

Evaluation of whole enzyme broth including fungal mycelia in the hydrolysis and fermentation of biomass was performed in 250 ml Erlenmeyer flasks essentially as described in the NREL protocol TP-510-42630. The used enzyme was an aseptically drawn final sample from a fermentation of the strain VTT-BR-C0022 on soybean hulls and sugarcane molasses (Fig. 9b). A sample of this whole broth was diluted 30-fold in 50 mM citrate pH 5.0, mixed and centrifuged. The protein quantified from the resulting supernatant (33.2 g/l) was considered for enzyme loading. The industrial pre-treated sugarcane straw substrate was washed twice with 5 volumes of ~80 °C deionized water to remove fermentation inhibitors, and the pH was adjusted to ~5.5 with NaOH. The biomass was then sterilized in an autoclave (120 °C, 20 min), and the dry-weight was quantified as previously. The pH of the biomass after autoclaving was ~5.1. 10 g of substrate (dry basis) were loaded into each flask, along with 5 ml of 500 mM citrate buffer at pH 5.1. The reaction was then filled up to 47.5 g with enzyme and water. An inoculum of *S. cerevisae* was prepared by cultivating overnight in 2% yeast extract, 1% peptone, and 5% dextrose (YPD5) medium. The cells were harvested by centrifugation (10 min, 1620*g*, 4 °C) and resuspended in sterile 0.9% NaCl. The cells were centrifuged again and finally resuspended in 10× Yeast Nitrogen Base (Sigma) to a final concentration of 20 g dry cell weight/l, and 2.5 ml of this suspension added to each flask to initiate fermentation with an initial pitch of 1 g dry cell weight/l. The flasks were purged with a sterile flow of nitrogen for 3 min, while adding the yeast inoculum and capped with bubble locks filled with 5 ml of sterile glycerol. The flasks were weighed after adding the yeast inoculum and at regular intervals thereafter. The weight loss of the bottles (due to CO_2_ evolution) was converted into ethanol concentration and glucan conversion.

### HPLC

High-performance liquid chromatography was used to quantify glucose, fructose, sucrose and xylose from cultivation and hydrolysis samples. The system used was Waters 1515 pump and 2414 detector at 50 °C, using a Rezex RFQ-Fast Acid H+ column at 85 °C and a flow rate of 0.8 ml/min of 5 mM H_2_SO_4_ as the mobile phase. For samples containing sucrose the temperature was lowered to 35 °C and the flow rate to 0.6 ml/min. Sugar standards were from Sigma Aldrich.

## Additional files

**Additional file 1: Table S1**. The industrial residues evaluated for cellulase production using *T. reesei* M44 in shake flask culture.

**Additional file 2: Figure S1**. Production of extracellular protein in bioreactor cultivations by *T. reesei* M44 on soybean hulls and VTT-BR-C0020 on soybean hulls and a feed comprising acid-invertsed sugarcane molasses.

**Additional file 3: Table S2**. The design of the vectors used to create strains VTT-BR-C0019, -C0020 and -C0022.

**Additional file 4: Table S3**. Primers used in the assembly of vectors pVTTBR43, pVTTBR54 and pVTTBR92, and for verification of inserts from genomic DNA.

## Abbreviations

MESP: minimum ethanol selling price
MUL: 4-methylumbelliferyl-β-d-lactopyranoside
CBH: cellobiohydrolase
EG: endoglucanase
SSF: simultaneous saccharification and fermentation
TRS: total reducing sugar
VVM: volume per volume per minute
PVDF: poyvinylidene fluoride
DO: dissolved oxygen
pNPG: 4-nitrophenyl-β-d-glucopyranoside
pNPX: 4-nitrophenyl-β-d-xylopyranoside
pNPA: 4-nitrophenyl-α-l-arabinofuranoside
CMC: carboxymethylcellulose
DNS: dinitrosalicylic acid
