# Supplementary material for: Development of a low-cost cellulase production process using Trichoderma reesei for Brazilian biorefineries
Source: Biotechnol Biofuels. 2017 Feb 2;10:30. doi: 10.1186/s13068-017-0717-0 (PMC5289010; doi:10.1186/s13068-017-0717-0)
Supplement: Supplementary file 1 — Additional file 1: Table S1. The industrial residues evaluated for cellulase production using T. reesei M44 in shake flask culture. [file 13068_2017_717_MOESM1_ESM.pdf]

Table S1 – The industrial residues evaluated for cellulase production using *T. reesei* M44 in shake flask culture. A residue was considered toxic if the strain presented no germination by 5 days in the presence of 30 g/l residue. Rheology was evaluated based on the maximal concentration not compromising agitation of the growth medium. Availability and price was assessed based on several Brazilian official sources. Induction was determined as the highest extracellular enzyme titer (g/l) achieved with each residue.

Industrial residue	Toxic	Rheology	Availability	Price	Induction
Sugarcane bagasse		+	++++	$	+
Sugarcane straw		+	++++	$	+
Pre-treated sugarcane straw	X	++	++++	$	N/A
Eucalyptus pulp		-	++++	$$$$	+
Cotton linter		-	++	$$$	+
Cotton hulls	X	++	++	$$	N/A
Corn silage	X	++	++	$$	N/A
Oat hulls	X	+++	+	$$	N/A
Soybean hulls		+++	+++	$$	+++
Brewer's spent grain		++	++	$$	+
Coffee hulls	X	++	++	$$	N/A
Sugarcane molasses		++++	+++	$$$	-
Milk whey		+++	+	$$$	++

		- = < 25 g/l		$ = < $50/t	+ = < 5 g/l
		+ = < 50 g/l		$$ = > $50	++ = 5 - 10 g/l
		++ = < 100 g/l		$$$ = > $150/t	+++ = > 10 g/l
		+++ = < 200 g/l		$$$$ = > $500/t	N/A = not applicable
		++++ = > 200 g/l			
