# Supplementary material for: Development of a low-cost cellulase production process using Trichoderma reesei for Brazilian biorefineries
Source: Biotechnol Biofuels. 2017 Feb 2;10:30. doi: 10.1186/s13068-017-0717-0 (PMC5289010; doi:10.1186/s13068-017-0717-0)
Supplement: Supplementary file 2 — Additional file 2: Figure S1. Production of extracellular protein in bioreactor cultivations by T. reesei M44 on soybean hulls and VTT-BR-C0020 on soybean hulls and a feed comprising acid-invertsed sugarcane molasses. [file 13068_2017_717_MOESM2_ESM.pdf]

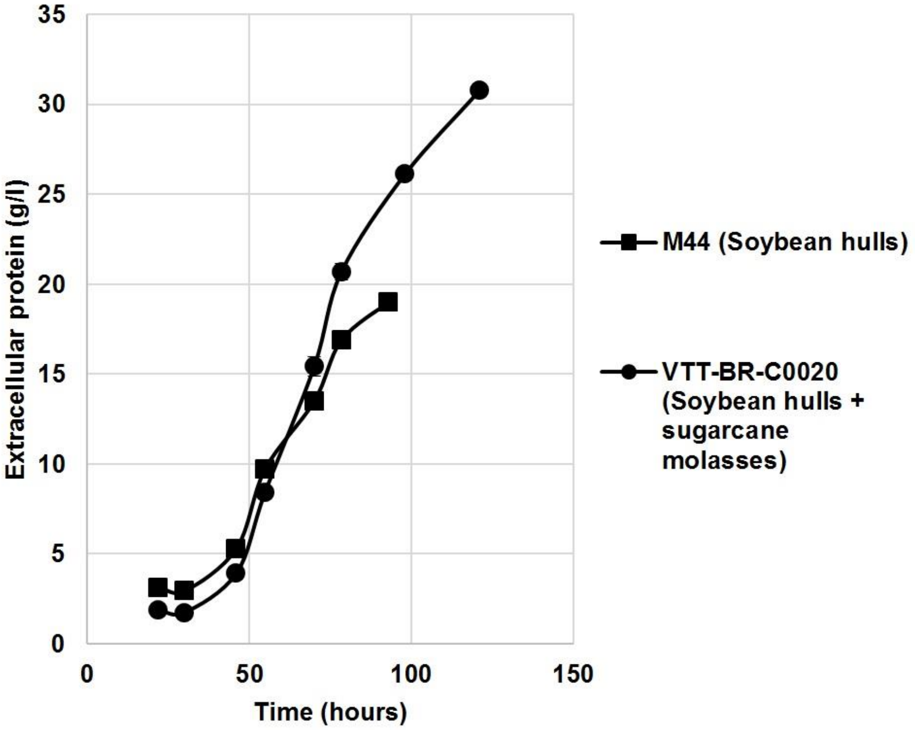

Figure S1

Extracellular protein production by strain M44 cultivated on soybean hulls and VTT-BR-C0020 cultivated on soybean hulls and sugarcane molasses. *Extracellular protein concentrations measured from culture supernatants of bioreactor cultivations of strain M44 on soybean hulls (13% in batch) and VTT-BR-C0020 on a batch medium comprising soybean hulls (13%), and with a feed of acid-inverted molasses.*
