# Supplementary material for: Development of a low-cost cellulase production process using Trichoderma reesei for Brazilian biorefineries
Source: Biotechnol Biofuels. 2017 Feb 2;10:30. doi: 10.1186/s13068-017-0717-0 (PMC5289010; doi:10.1186/s13068-017-0717-0)
Supplement: Supplementary file 3 — Additional file 3: Table S2. The design of the vectors used to create strains VTT-BR-C0019, -C0020 and -C0022. [file 13068_2017_717_MOESM3_ESM.pdf]

Table S2 – The design of the vectors used to create strains VTT-BR-C0019, -C0020 and –C0022 (pVTTBR43, pVTTBR54 and pVTTBR92, respectively). NNNN = 5'->3' overlap left by BsaI-digestion and used for vector assembly. Start codon. All vectors assembled into pUC57-Kan derived backbone, with MssI-sites on either side of the overlaps GGGT / CCGC

Vector	NNNN	Promoter	NNNN	Gene	NNNN	Terminator	NNNN	Marker	NNNN	6th frag.	NNNN
**pVTTBR43**	GGGT	*pdc1*[a]	CATG	*xyr1_V821F*[a]	TGAA	*pdc1*[a]	ATGA	*hph_amdS*[c]	CCGC		
**pVTTBR54**	GGGT	*xyn1*[b]	CATG	*TeCel3A*[a]	TGAA	*xyn1*[b]	ATGA	*bar*[c]	GAGA	CBHI-3'	CCGC
**pVTTBR92**	GGGT	*pdc1*[a]	CATG	*ace2*[b]	TGAA	*pdc1*[a]	ATGA	*thi4*[b+]	GAGA	CBHI-3'*	CCGC

Fragment sources: [a] = GenScript Ltd., [b] = *T. reesei* M44 genomic DNA ([b+] = mutated), [c] = pre-existing plasmid. *later replaced with *suc1* amplified from *A. niger* VTT-D-77050 genomic DNA.
