# Supplementary material for: Development of a low-cost cellulase production process using Trichoderma reesei for Brazilian biorefineries
Source: Biotechnol Biofuels. 2017 Feb 2;10:30. doi: 10.1186/s13068-017-0717-0 (PMC5289010; doi:10.1186/s13068-017-0717-0)
Supplement: Supplementary file 4 — Additional file 4: Table S3. Primers used in the assembly of vectors pVTTBR43, pVTTBR54 and pVTTBR92, and for verification of inserts from genomic DNA. [file 13068_2017_717_MOESM4_ESM.pdf]

Table S3 – Primers used in the assembly of vectors pVTTBR43, pVTTBR54 and pVTTBR92, and for verification of inserts from genomic DNA.

Primer code	***Primer name***	***5'-3' Sequence***
BR63	pdc_F	GCGCCTCAGAGTGTCGTC
BR89	xyr1_ins_R	GGACAATGGCAGATGCTTGG
BR103	TeCel3A_GG_R	AATGGTCTCTTTCAAATTCCAGGGTATGGCTTAAGG
BR104	TeCel3A_mut_F	CTGGGCAGGTCCCCCGATGCGGGCAGAAAC
BR105	TeCel3A_mut_R	GTTTCTGCCCGCATCGGGGGACCTGCCCAG
BR106	TeCel3A_GG_F	AATGGTCTCACATGAGGAACGGGTTGCTCAAGGTC
BR117	pXyn11B_GG_F	AATGGTCTCAGGGTACAGCATATTTCGTTGGCTGGCAG
BR118	pXyn11B_GG_R	AATGGTCTCACATGATGATTATTTGTGCGTGTTTTCC
BR119	tXyn11B_GG_F	AATGGTCTCATGAAGTTCTGTTGATGTTGACTTGGAGTGGATG
BR120	tXyn11B_GG_R	AATGGTCTCATCATGTGGCGAGGATCTAGCGGGAG
BR133	pGdpA_GG_F	AATGGTCTCAATGATCTCCTTAGCTCTG
BR134	tTrpC_GG_R	AATGGTCTCAATGATCTCCTTAGCTCTG
BR143	pXyn11B_F	CGTAAAACTGAGACAGCAAGCTC
BR145	tXyn11B_R	GTCTGTCAAGTTAACTCACTAACC
BR190	ace2_GG_F	AATGGTCTCACATGGACCTCCGGCAAGC
BR191	ace2_GG_R	AATGGTCTCTTTCACTTCAGCAGTCTGGCACTG
BR192	suc_F	CACGCGTTCAACATGGACAAG
BR193	suc_R	GTATCGGGAAGGGAAAACAACTG
BR213	thi4_mut_F	GGATAATACCAGCGAAAGGGTCATGCTCCTCCCCCTTTTC
BR214	thi4_mut_R	GAAAAGGGGGAGGAGCATGACCCTTTCGCTGGTATTATCC
BR215	thi4_GG_R	AATGGTCTCATCTCTTCTGAGGTGCCGATCC
BR216	thi4_GG_F	AATGGTCTCAATGAGTCCACCACAACTTTCTTAGC
BR259	CBHflank_suc_InF_F	TTCCCTTCCCGATACAGCGATCAACAACCGTGC
BR260	CBHflank_suc_InF_R	CATGTTGAACGCGTGAGCTGCGGCTAGGACGTC
